# Retinal drusen in patients with chronic myeloproliferative blood cancers are associated with an increased proportion of senescent T cells and signs of an aging immune system

**DOI:** 10.18632/aging.203803

**Published:** 2021-12-26

**Authors:** Charlotte Liisborg, Vibe Skov, Lasse Kjær, Hans Carl Hasselbalch, Torben Lykke Sørensen

**Affiliations:** 1 Department of Ophthalmology, Zealand University Hospital, Roskilde 4000, Denmark; 2 Faculty of Health and Medical Sciences, University of Copenhagen, Copenhagen 2200, Denmark; 3 Department of Hematology, Zealand University Hospital, Roskilde 4000, Denmark

**Keywords:** age-related macular degeneration, drusen, myeloproliferative neoplasms, chronic low-grade inflammation, immunosenescence, T cells

## Abstract

The cause of age-related macular degeneration (AMD) is unknown, but evidence indicates that both innate and adaptive immunity play a role in the pathogenesis. Our recent work has investigated AMD in patients with myeloproliferative neoplasms (MPNs) since they have increased drusen and AMD prevalence. We have previously found increased levels of chronic low-grade inflammation (CLI) in MPN patients with drusen (MPNd) compared to MPN patients with normal retinas (MPNn). CLI and AMD are both associated with aging, and we, therefore, wanted to study immunosenescence markers in MPNd, MPNn, and AMD. The purpose was to identify differences between MPNd and MPNn, which might reveal novel information relevant to drusen pathophysiology and thereby the AMD pathogenesis. Our results suggest that MPNd have a T cell differentiation profile resembling AMD and more effector memory T cells than MPNn. The senescence-associated-secretory-phenotype (SASP) is associated with effector T cells. SASP is thought to play a role in driving CLI seen with advancing age. Senescent cells with SASP may damage healthy tissue, including the eye tissues affected in AMD. The finding of increased effector cells in MPNd could implicate a role for adaptive immunity and senescent T cells together with increased CLI in drusen pathophysiology.

## INTRODUCTION

The exact cause of the eye disease age-related macular degeneration (AMD) is unknown, but the most significant risk factor is aging [1]. A study estimates that nearly 200 million people globally have AMD. These numbers are expected to rise to nearly 300 million by 2040 due to increased life expectancy [2]. AMD is a debilitating disease affecting central visual acuity and complicates everyday activities such as reading, driving, and recognizing faces. There is an urgent need for a better understanding of the condition and better treatment options.

Age-related macular degeneration is divided into early-, intermediate- and late AMD. Late-stage AMD can take two forms: the fast-developing neovascular AMD (nAMD) and the more slowly progressing atrophic form, geographic atrophy (GA).

A characteristic of all stages of AMD is the presence of small deposits of lipids and proteins (drusen) lying below or above the retinal pigment epithelium (RPE) of the retina [3]. Why we develop drusen is not entirely understood, but they occur with increasing age in most people, and the risk of developing late AMD increase with the number and size of drusen [4, 5]. The buildup of cellular damage characterizes the aging process, and the declining functions of the repair mechanisms can lead to the buildup of cellular defects or debris [6]. The current understanding of drusen pathophysiology is a process initiated locally and starts with the accumulation of debris in Bruch’s membrane (BM) and the following thickening of this layer. The debris buildup occurs partly by accumulating waste products from the visual cycle and nutrients and waste products from the bidirectional transport between the choroid, BM, RPE, and photoreceptors. With age, the thickening of the BM seems to decrease the function of the bidirectional flow, and an accumulation of lipids is seen, resulting in drusen formation. The RPE cells are suspected of contributing to this process and the subsequent complement activation and induction of reactive oxygen species (ROS) [7, 8]. In a proposed two-level hypothesis, this random accumulation of debris is the “first step,” and the systemic inflammatory response to this damage is the second. Both steps are thought to be necessary to develop AMD [8].

Our recent work investigated AMD in patients with Philadelphia chromosome-negative myeloproliferative neoplasms (MPN) [9]. The MPNs are a group of closely related hematological cancers named essential thrombocythemia (ET), polycythemia vera (PV), and primary myelofibrosis (PMF). They are thought to evolve in a biological continuum from early-stage ET to PV to PMF [10, 11]. The diseases are characterized by acquired driver mutations in the *JAK2, MPL,* and *CALR* genes, resulting in excessive production of myeloid cells, overproduction of inflammatory markers, and a massive symptom burden [12].

The rationale for investigating patients with MPNs is that we have found an increased prevalence of AMD, including an increased prevalence of drusen in these patients [13, 14]. The MPNs do not only have a higher prevalence of drusen and AMD, but drusen also show up earlier, and significantly more younger people have drusen than the background population [14]. We have also shown that the drusen prevalence in MPNs was associated with an increased level of chronic low-grade inflammation (CLI), and we also found evidence of a dysregulated complement system. In this recent work, we proposed to use MPNs as a “Human Inflammation Model” of drusen development. The CLI triggers drusen formation, leading to more CLI, creating a self-perpetuating vicious cycle, increasing the risk of developing late AMD. This idea challenges the current theory of drusen pathophysiology happening at least initially locally.

Both CLI and AMD are associated with aging. In AMD, retinal aging is observed, and in peripheral blood of these patients, accelerated T cell differentiation and elevated aging markers have been found [15], but we do not know the role of the aging peripheral immune system on AMD. Patients with MPNs provide a unique opportunity to investigate immune aging in patients with and without drusen and evaluate if patients with MPNs also show signs of accelerated immune aging.

Numerous studies of the normal aging process have identified changes in the immune system with age. These changes are often termed “immunosenescence,” and one of the most notable changes is that the proportion of naïve CD8 T cells decreases. In contrast, the memory T cell pool increase, altering the cytokine profile since the different T cells secrete various cytokines, which alter functions of T cells such as cytokine production and T cell proliferation. Studies indicate that senescent T cells are synonymous with effector T cells [16–18], and therefore, senescent T cells accumulate with age [19–21].

The CD4 T cell compartment shows smaller age-related changes than the CD8 compartment [19, 20]. Other recognized changes with age are the loss of the co-receptors for T cell stimulation CD27 and CD28 [21], the upregulation of cytolytic activity, and an increase in markers commonly associated with natural killer cells (NKRs) [22–25]. Also, a chronic pro-inflammatory milieu is associated with aging, often termed “inflammaging” [26].

**Figure 1:**
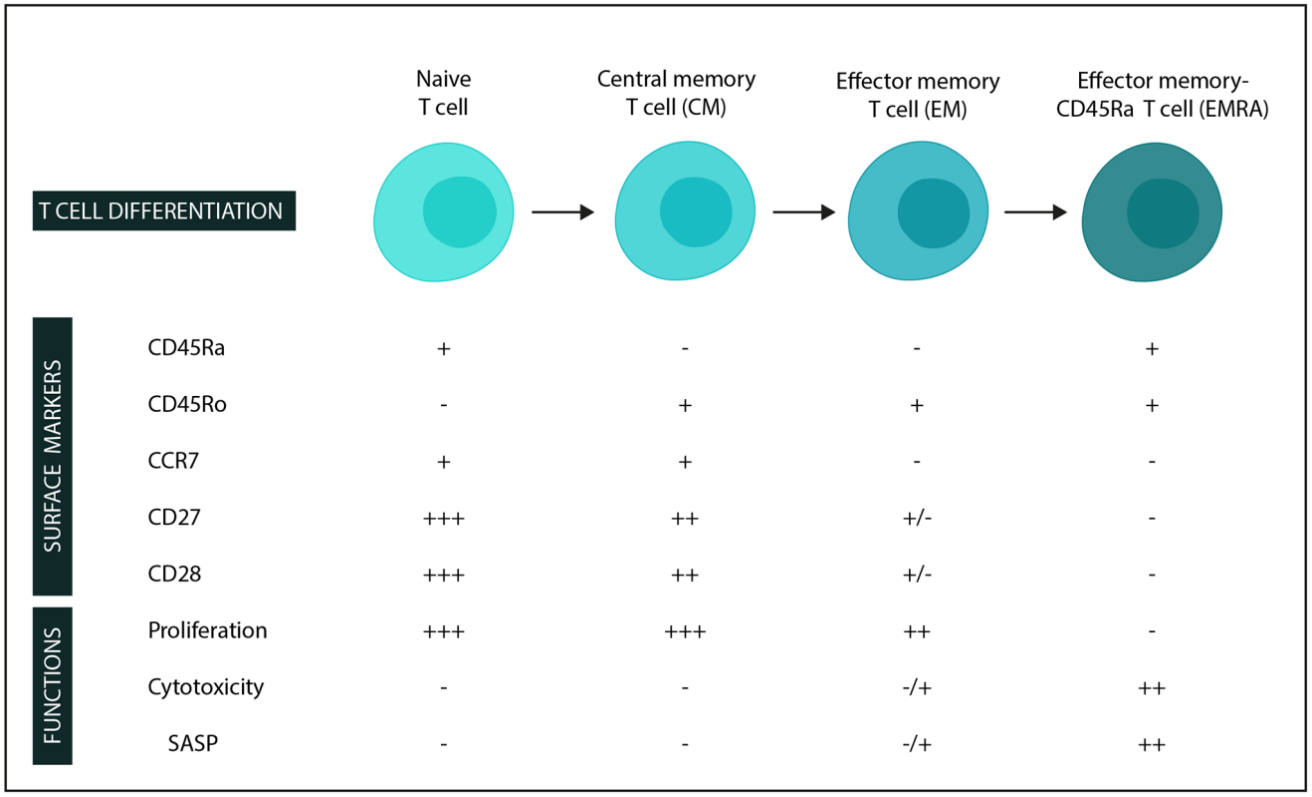
**Surface markers and functions in stages of T-cell differentiation (in the CD4 and CD8 compartments).** −: low expression/not expressed/function not present. +: expressed/function present, and additional +’s: higher expression/function. Abbreviation: SASP: Senescence-associated secretory phenotype.

With this study, we wanted to investigate markers of immunosenescence in patients with MPN and MPN subtypes. We specifically evaluated CD4+ and CD8+ T cell differentiation (naïve, central memory (CM), effector memory (EM), and effector memory CD45Ra+ cells (EMRA) – Figure 1 [16, 17, 27]). Additionally, we assessed the loss of the costimulatory markers CD27 and CD28 and the expression of an NKR CD56+ used as a marker for T cell aging. We also wanted to compare patients with MPNs and drusen (MPNd) with patients with MPNs and normal retinas (MPNn) and finally to compare these results with patients having AMD. The purpose was to identify differences between patients with and without drusen and how these patients resemble patients with AMD. This may reveal information that could be relevant for the pathogenesis of AMD.

## RESULTS

### Study population

We included 123 patients in the study. Four patients were excluded post hoc. One patient because of a high CRP, implying an ongoing acute immune response, two patients because they fulfilled the criteria of having GA; and one was excluded because the flow cytometric analyses failed. The result was 119 included patients: 29 nAMD, 28 iAMD, 35 MPNd, and 27 MPNn. The patients are the same as in our previous work, and patient characteristics (Table 1) are therefore identical [9].

**Table 1:** Patient characteristics.

	**nAMD (*n* = 29)**	**iAMD (*n* = 28)**	**MPNd (*n* = 35)**	**MPNn (*n* = 27)**	***p*-value**
**Demographics**					
Age, years, median (IQR)	77 (71–82)	73 (68–76)	72 (65–76)	69 (62–74)	**<0.001^a^**
Sex					0.28^b^
*Males, n (%)*	12 (41)	10 (36)	20 (57)	10 (37)	
*Females, n (%)*	17 (59)	18 (64)	15 (43)	17 (63)	
**Lifestyle factors**					
Smoking, *n* (%)					0.83^c^
*Never*	12 (41)	12 (43)	16 (46)	11 (41)	
*Former*	13 (45)	13 (46)	18 (51)	14 (52)	
*Current*	4 (14)	3 (11)	1 (3)	2 (7)	
Body mass index, mean (95%CI)	26 (24–27)	25 (24–27)	25 (24–27)	27 (25–29)	0.48^d^
Alcohol consumption, units per week, median (IQR)	2 (0–7)	3 (0–7)	7 (2–14)	2 (0–8)	**0.0036^a^**
**Comorbidities**					
Cardiovascular disease, *n* (%)	4 (14)	5 (18)	6 (17)	6 (22)	0.89^c^
Hypertension, *n* (%)	13 (45)	8 (29)	18 (51)	17 (63)	0.075^b^
Hypercholesterolemia, *n* (%)	4 (14)	2 (7)	3 (9)	2 (7)	0.82^c^
Type 2 diabetes, *n* (%)	2 (7)	1 (4)	2 (6)	0 (0)	0.76^c^
**MPN diagnosis (MPN patients only)**					0.082^b^
Essential thrombocythemia, *n* (%)	−	−	6 (17)	11 (41)	
Polycythemia vera, *n* (%)	−	−	26 (74)	13 (48)	
Pre-PMF, *n* (%)	−	−	0 (0)	1 (4)	
Primary myelofibrosis, *n* (%)	−	−	3 (9)	2 (7)	
**Mutation status (MPN patients only)**	−	−			0.43^c^
*JAK2V617F, n (%)*	−	−	31 (91)	22 (82)	
*CALR* mutation, *n* (%)	−	−	1 (3)	3 (11)	
*MPL* mutation, *n* (%)	−	−	1 (3)	0 (0)	
Triple-Negative, *n* (%)	−	−	1 (3)	2 (7)	

Significant *p*-values are shown in bold. Statistical comparisons between groups: ^a^Kruskal Wallis test, ^b^Pearson's Chi-squared test, ^c^Fischer’s exact test, ^d^One-way ANOVA. Abbreviations: AMD: age-related macular degeneration; nAMD: neovascular AMD; iAMD: intermediate AMD; MPN: myeloproliferative neoplasms; MPNd: Patients with MPN and drusen; MPNn: patients with MPN and normal retinas; IQR: interquartile range; PV: polycythemia vera; ET: essential thrombocythemia; PreMF: pre-myelofibrosis; PMF: primary myelofibrosis; *JAK2V617F*: mutation in the *JAK2* gene; *CALR*: calreticulin gene; *MPL*: *MPL* gene; the gene encoding the thrombopoietin receptor.

Patients with nAMD had a median age of 77 (IQR: 71–82) years, significantly older than iAMD (73 years, IQR: 68–76, *p* = 0.034) MPNd (72 years, IQR: 65–76, *p* = 0.0040) and MPNn (69 years, IQR: 62–74, *p* < 0.001). No differences were found between the groups regarding sex, smoking habits, body mass index, and comorbidities. The MPNd group had a higher median alcohol consumption of 7 units per week than nAMD (2, IQR: 0–7, *p* < 0.001), iAMD (3, IQR: 0–7, *p* = 0.019), and MPNn (2, IQR: 0–8, p0.021). We found no influence of alcohol consumption on outcomes reported in the following sections. The distribution of subtypes of MPNs was 39 PV-, 17 ET-, and six PMF patients. Most patients with MPN had the *JAK2V617F*-mutation (82%), fewer *CALR* (6.2%), and *MPL* (1.5%). We detected no difference in JAK2V617F allele burden between the two MPN groups (*p* = 0.088), but it seemed that MPNn had a lower allele burden of 17% compared to 33% for MPNd. We also subdivided allele burden into four groups of 0–25%, >25–50%, >50%–75%, and >75%, and no difference in distribution between the two groups was seen (*p* = 0.61). There were no differences between the MPN groups regarding treatment with hydroxyurea (HU) (*p* = 0.99). All received acetylsalicylic acid or other anticoagulant therapy. The distribution of patients receiving statins was similar across all groups (*p*-value = 0.58).

### CD4+ and CD8+ T cells

**Figure 2:**
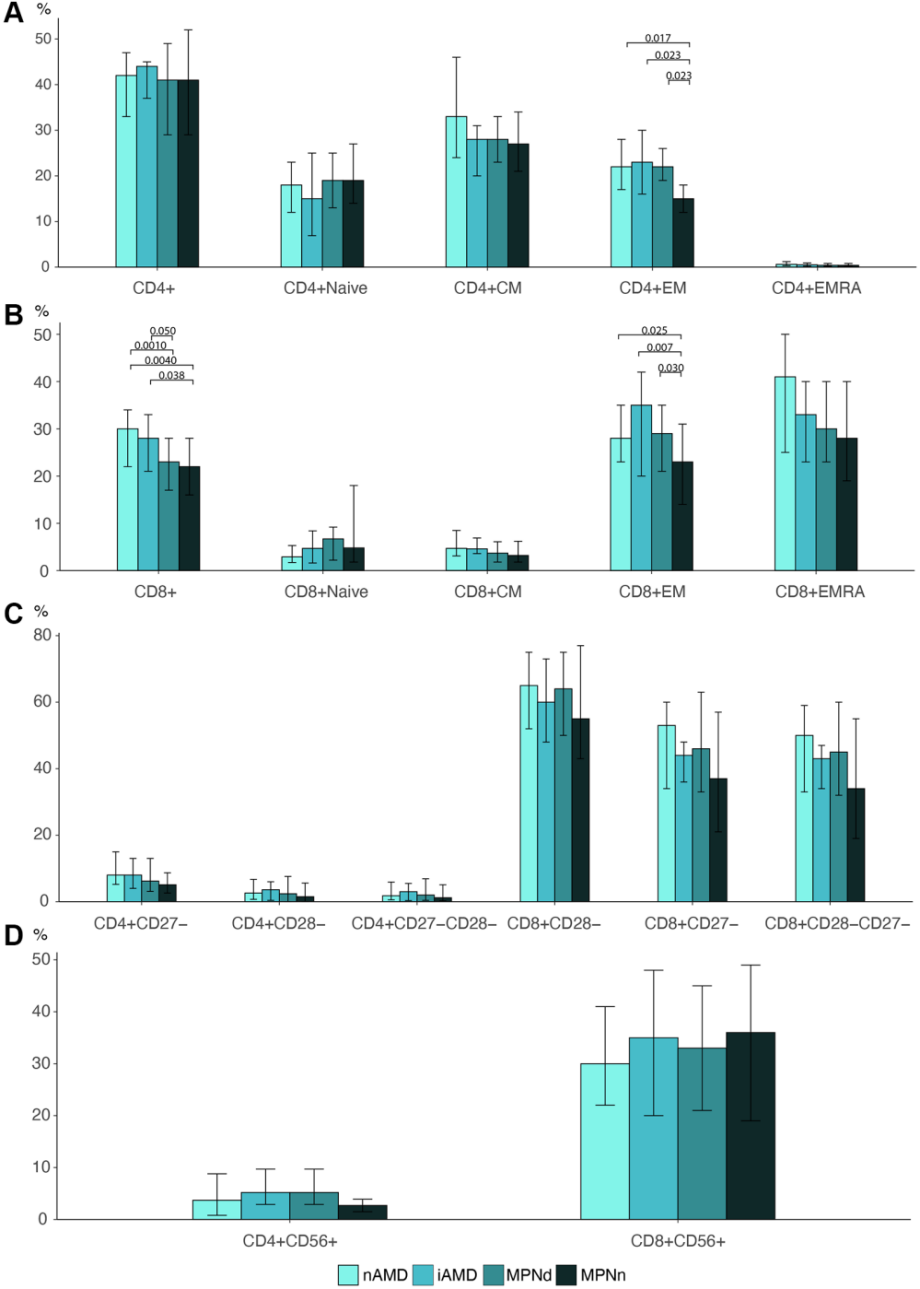
Barplots of (**A**) CD4 T cell differentiation profile. (**B**) CD8 T cell differentiation profile. (**C**) Loss of costimulatory markers in CD4 and CD8 T cells. (**D**) CD56 expression in CD4 and CD8 cells Statistically significant *p*-values are shown above bar plots. Statistical comparisons between groups: Kruskal Wallis test or robust linear regression if the outcome were age-dependent and Wilcoxon rank-sum test for multiple comparisons. Abbreviations: nAMD: neovascular AMD; iAMD: intermediate AMD; MPN: myeloproliferative neoplasms; MPNd: Patients with MPN and drusen; MPNn: patients with MPN and normal retinas; Naïve: naïve T cells; CM: central memory T cells; EM: effector memory T cells; EMRA: effector memory CD45Ra+ T cells.

Patients with iAMD had a lymphocyte percentage of 15% (CI: 13–18), significantly higher than 11% (CI: 8.8–13) in AMD (*p* = 0.0050), 8.8% (CI: 7.3–10) in MPNd (*p* < 0.001) and 9.9% (CI: 8.6–11) in MPNn (*p* < 0.001). No difference was found in the CD4+ T cell percentage between the groups (*p* = 0.86), but AMD and iAMD had a statistically significant higher percentage of CD8+ T cells (30% (IQR: 22–34) and 28% (IQR: 21–33)) than the MPNd and MPNn groups (23% (IQR: 17–28) and 22% (IQR: 16–28)) (nAMD- MPNd: *p* = 0.0010, nAMD-MPNn: *p* = 0.0040, iAMD-MPNd: *p* = 0.050, iAMD-MPNn: *p* = 0.038) (Figure 2B). We did not find any differences between groups in CD4/CD8 ratio or the CD4+CD8+ double-positive cells percentage (*p* = 0.68 and *p* = 0.44) (data not shown).

### T cell differentiation

We investigated the T cell differentiation profile (Naïve-, CM, EM, and EMRA T cells) in all groups (Figure 2A and 2B). We found a statistically significant difference in the distribution of EM T cells in both the CD4 and CD8 T cell compartment. In MPNn patients, EM cells accounted for 15% (IQR: 12–18) of the total CD4+ cells, significantly lower than 22% (IQR: 19–26) in MPNd (*p* = 0.023), 23% (IQR: 16–30) in iAMD (*p* = 0.023), and 22% (IQR: 17–28) in nAMD patients (*p* = 0.017). The same was observed for CD8 T cells where EM cells comprised 23% (IQR: 14–31) of CD8 T cells in MPNn compared to 29% (IQR: 21–35) in MPNd (*p* = 0.030) 35% (IQR: 20–42) in iAMD (*p* = 0.0070) and 28% (IQR: 23–35) in nAMD patients (*p* = 0.025).

### Costimulatory markers CD27 and CD28 and CD56+ expression

Although there was a tendency for MPNn patients to lose less of the differentiation markers CD27 and CD28 compared to the other groups, the differences were not statistically significant (Figure 2C).

We did not observe any differences in the expression of the aging marker CD56 between any of the groups (Figure 2D).

### MPN biological continuum

**Figure 3:**
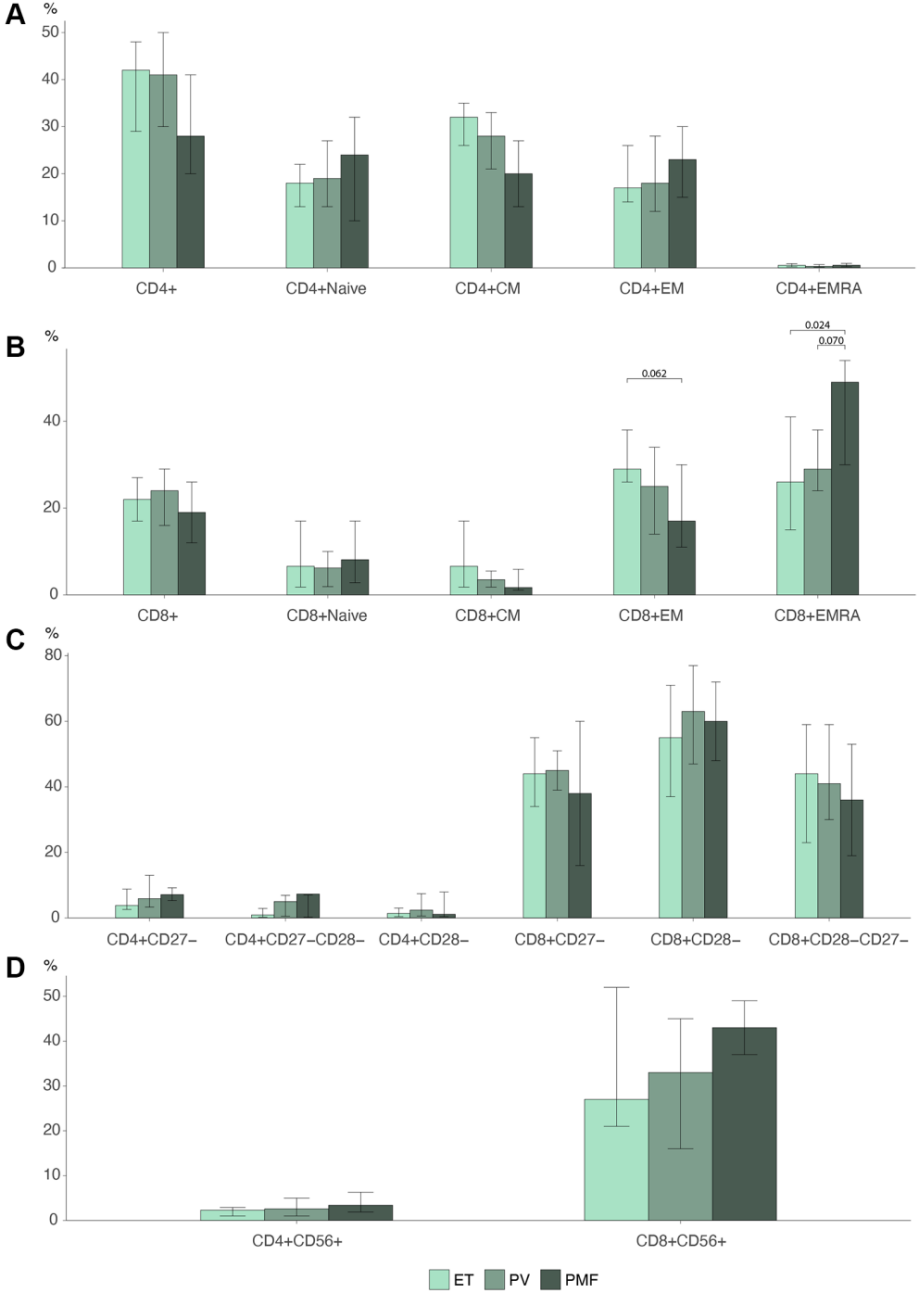
Barplots MPN subtypes of (**A**) CD4 T cell differentiation profile (**B**) CD8 T cell differentiation profile. (**C**) Loss of costimulatory markers in CD4 and CD8 T cells. (**D**) CD56 expression in CD4 and CD8 cells Statistically significant *p*-values are shown above bar plots. Statistical comparisons between groups: Kruskal Wallis test or robust linear regression if the outcome were age-dependent and Wilcoxon rank-sum test for multiple comparisons. Abbreviations: ET: essential thrombocythemia; PV: polycythemia vera; PMF: primary myelofibrosis; Naïve: naïve T cells; CM: central memory T cells; EM: effector memory T cells; EMRA: effector memory CD45Ra+ T cells.

We investigated differences in the biological continuum in MPNs from ET to PV to PMF (Figure 3).

The lymphocyte percentage decreased over the biological continuum. ET patients had 11% (CI: 9.4–13) lymphocytes compared to 8.9% (CI: 7.6–10) in PV patients (*p* = 0.043) and 6.7% (CI: 4.4–10) in PMF patients (*p* = 0.022) (data not shown). PMF patients had a percentage of terminally differentiated EMRA cells in the CD8 compartment of 49% (IQR: 30–54), significantly higher than 26% (IQR: 15–41) in ET patients (*p* = 0.024) and near significantly higher than 29% (IQR: 24–38) in PV patients (*p* = 0.070) (Figure 3B). We found no significant differences in the CD4 compartment (Figure 3A). PMF patients correspondingly seemed to have a lower percentage of EM cells, 17% (IQR: 11–30), than PV 25% (IQR: 14–34) (*p* = 0.22) and ET 26% (IQR: 26–38) (*p* = 0.062), but this was not statistically significant.

The expression of CD56 seemed to rise over the biological continuum (Figure 3D), but this was not statistically significant (CD4 *p* = 0.38, CD8 *p* = 0.19). There were no differences between the groups in the loss of differentiation markers CD27 and CD28 (Figure 3C).

### Immunosenescence plots

**Figure 4:**
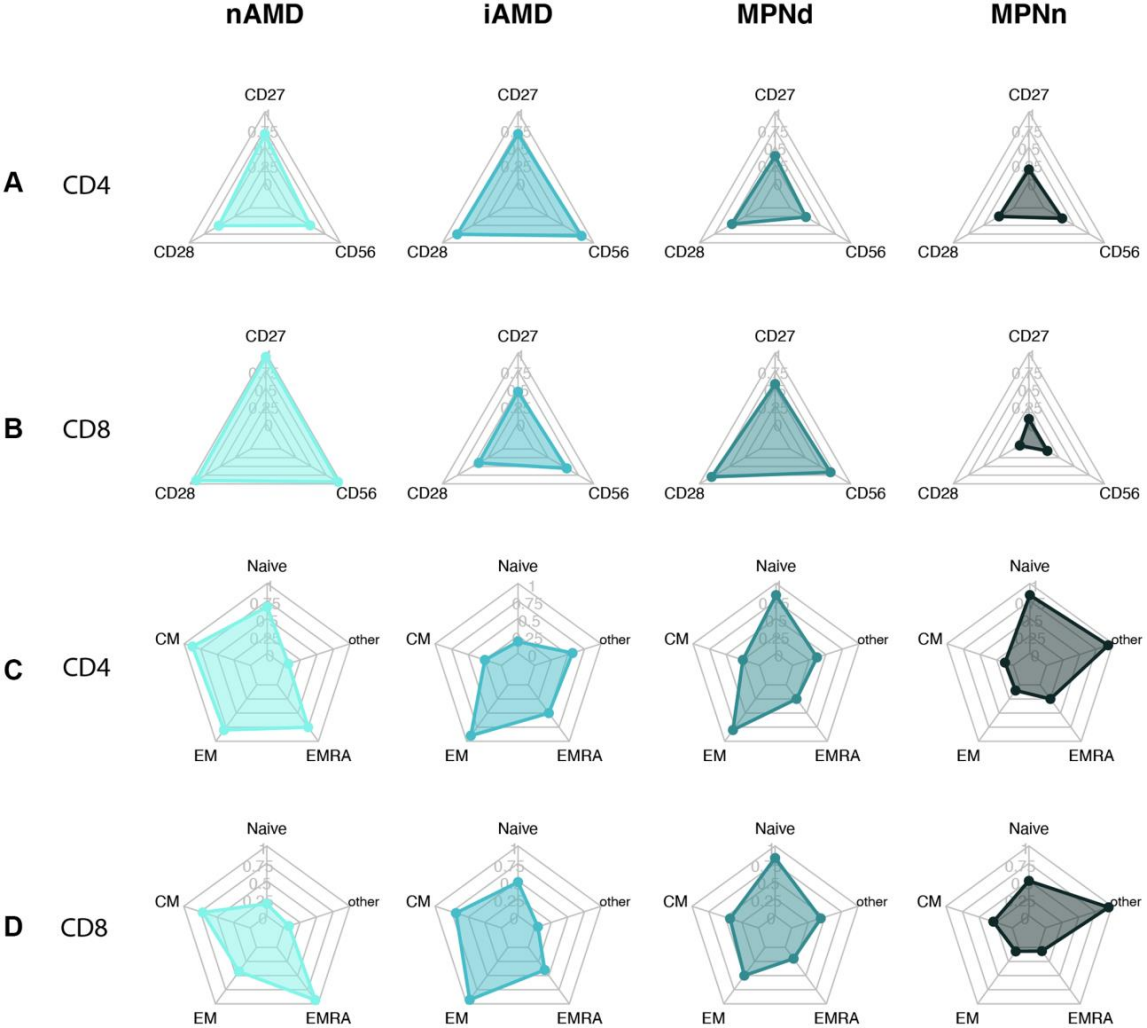
Radarplots of (**A**) CD4+ T cell with loss of CD27 and CD28 and CD56 expression. (**B**) CD8+ T cell with loss of CD27 and CD28 and CD56 expression. (**C**) CD4+ T cell differentiation profile. (**D**) CD8+ T cell differentiation profile. A more senescent profile is characterized by loss of CD27 and CD28, more CD56, and more terminally differentiated cells (EM and EMRA). Abbreviations: nAMD: neovascular AMD; iAMD: intermediate AMD; MPN: myeloproliferative neoplasms; MPNd: Patients with MPN and drusen; MPNn: patients with MPN and normal retinas Naïve: naïve T-cells; CM: central memory T cells; EM: effector memory T cells; EMRA: effector memory CD45Ra positive T cells; other: includes intermediate subsets of T cells not belonging to Naïve, CM, EM, or EMRA.

**Figure 5:**
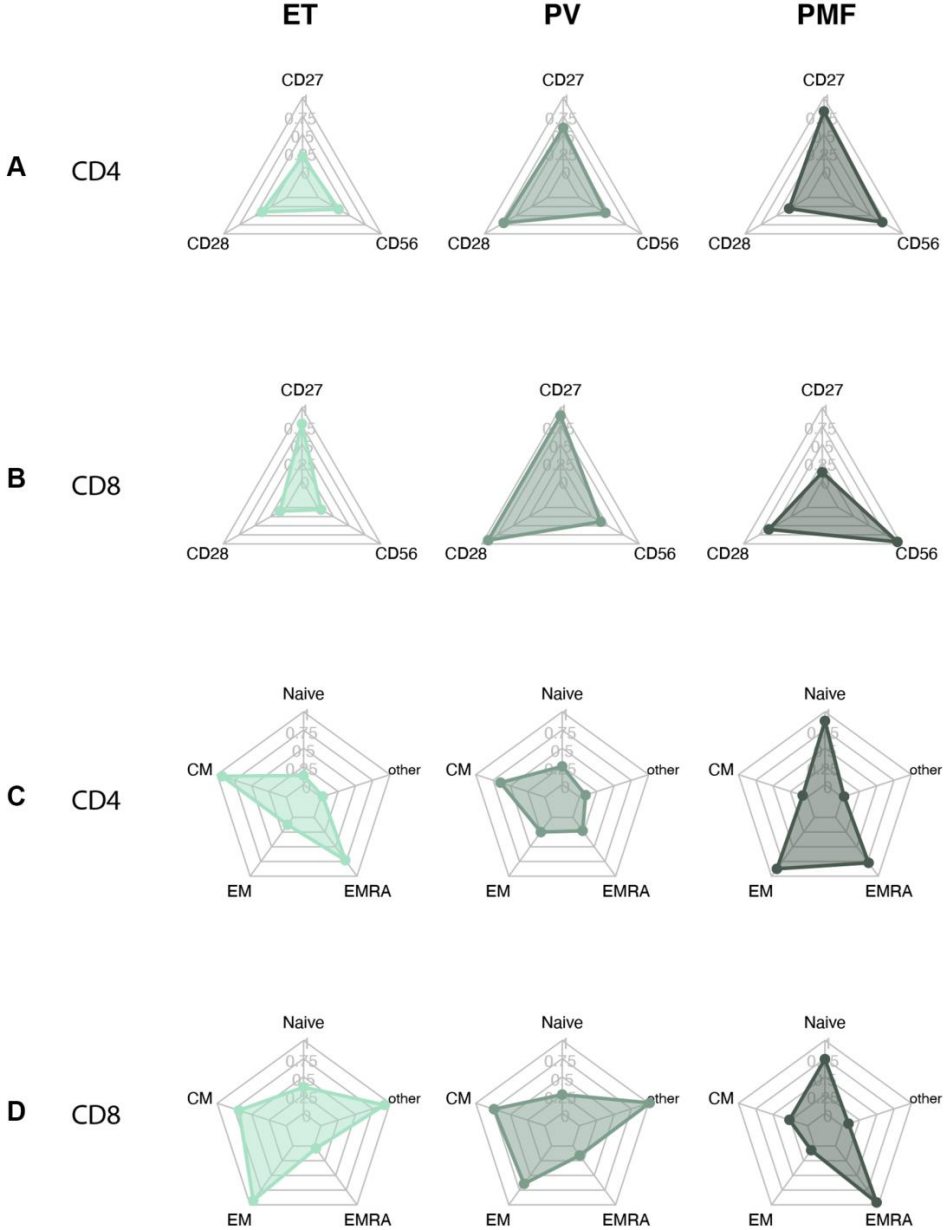
Radarplots MPN subtypes of (**A**) CD4+ T cell with loss of CD27 and CD28 and CD56 expression. (**B**) CD8+ T cell with loss of CD27 and CD28 and CD56 expression. (**C**) CD4+ T cell differentiation profile. (**D**) CD8+ T cell differentiation profile. A more senescent profile is characterized by loss of CD27 and CD28, more CD56, and more terminally differentiated cells (EM and EMRA). Abbreviations: ET: essential thrombocythemia; PV: polycythemia vera; PMF: primary myelofibrosis. Naïve: naïve T-cells; CM: central memory T cells; EM: effector memory T cells; EMRA: effector memory CD45Ra positive T cells; other: includes intermediate subsets of T cells not belonging to Naïve, CM, EM, or EMRA.

To visualize our results, we created radar plots of the different T cell subsets and differentiation markers to evaluate the resemblance among groups further. MPNn patients stood out in most plots compared to the other groups (Figure 4). The MPNd, AMD, and iAMD patients seemed to have a more senescent profile, with more loss of costimulatory markers, higher CD56 expression, and more terminally differentiated T cells. In Figure 5, we compare MPN subtypes. ET and PV patients looked more alike with minor shape changes, but PMF patients stood out with a more senescent profile.

## DISCUSSION

Age-related macular degeneration is a common and debilitating disease affecting millions worldwide. Today treatment for nAMD is regular anti-vascular endothelial growth factor eye injections, which may slow the disease and prevent further vision loss. We have no treatment options for the early- or intermediate stages of AMD or the late atrophic form, GA. A better understanding of the underlying disease mechanisms can lead us to new target areas aiding the development of new therapies. Especially information on drusen pathophysiology would be of great importance and may eventually lead to treatment options for the disease in earlier stages.

We have investigated the immune systems of patients with MPNs because they have an increased prevalence of drusen and late AMD [14]. We have published several differences between MPN patients with and without drusen. The MPNd have a higher level of CLI and signs of a dysregulated complement system [9]. In this study, we investigated immunosenescence markers, and the results suggest that MPNd have accelerated T cell differentiation with more effector T cells than MPNn and a similar differentiation profile to AMD and iAMD patients. We observed a tendency for a smaller loss of costimulatory markers in MPNn, but the results were not statistically significant. The CD56 expression on CD28 negative cells has previously been found to be increased in patients with AMD compared to healthy individuals [28]. We found no difference in the expression of the NKR CD56 between the groups. However, the increase in CD56 expression plateaus in the seventh decade of life. All the groups’ median ages were at the end of the seventh- or in the eighth decade and could explain the similar CD56 expression [22]. Finally, we also observed a smaller percentage of CD8 T cells in patients with MPN than AMD patients, which may reflect accelerated immune aging in these patients, since the overall CD8 T cell reservoir decreases with age [29].

One of the most notable and recognized changes with age is the depletion of the CD8 naïve T cell pool while the memory T cell pool increase [19]. After thymic involution around puberty, the thymic output of naïve T cells progressively declines, and the cells are hereafter maintained primarily by proliferation from the existing pool [20, 30]. The naïve CD4 T cell pool number remains relatively stable during adulthood, but a similar accumulation of memory and effector cells occurs for these cells too, although later in life than for the CD8 compartment [20, 21].

Naïve T cells are activated and differentiate to become central memory or effector memory cells. We define the differentiated cells according to their markers (CD45Ra, CD45Ro, and CCR7) [21, 31, 32] and function (Figure 1). Differentiated cells also gradually lose the costimulatory molecules CD27 and CD28, which play an important part in T cell activation, such as cytokine production and stimulating cell proliferation [21, 27, 33, 34]. The CD8 cells lose CD28 first and then CD27, while the opposite is the case for CD4 cells, which lose CD27 first, followed by CD28 loss [21]. Further, the upregulation of cytolytic activity and markers commonly associated with NK cells are seen, giving the cells a cytotoxic capability. One example is CD56, as investigated in this study, one of the best-described markers of T cell aging [22–25].

Evidence indicates that senescent T cells are synonymous with effector T cells [16–18]. So, with age, senescent T cells accumulate [19–21]. DNA damage due to stress factors as oxidative- and replicative stress and the following repair mechanisms can induce senescence [35, 36]. Also, infection and inflammation drive T cells to senescence [37], and CLI is associated with progressive T cell differentiation [21]. An example of an infection that drives CD8 senescent cells to accumulate is cytomegalovirus (CMV) [38], and seropositivity for CMV is interestingly also associated with an increased risk of AMD [39].

Senescent cells do not proliferate but are active and secrete cytokines, chemokines, and cytotoxic granules referred to as the senescence-associated secretory phenotype (SASP) initially established in fibroblast but also shown in other cells, including T cells [16, 40, 41]. The SASP is thought to play a role in driving inflammaging [42]. The cells acquiring SASP may also be beneficial in preventing, for instance, cancer but can also become dysregulated and accelerate inflammaging [43]. The characteristics of senescent T cells are highly inflammatory and cytotoxic cells that secrete cytotoxic mediators and may damage healthy tissue [27]. Senescent cell accumulation may underlie many age-related diseases such as cardiovascular- [44–46], autoimmune- [47], and neurodegenerative diseases [48, 49].

Interestingly, lymphocytes, including CD8 T cells, have been observed in the choroid of eyes from AMD patients [50–52], and CD8 positive cells are more abundant in the macular choroid of patients with drusen [50]. In this study, the finding of increased effector cells in MPNd could be supportive of a role of CD8 T cells in the drusen/AMD pathogenesis. We do not know, though, if the increase in CD8+ T effector cells preceded the appearance of drusen or is a result of drusen presence. Either way, the presence of CD8 effector cells could potentially harm the choroid and, hereafter, the BM, RPE, and photoreceptors. Previous studies have shown that activated T-cells can modify the chemokine profile [53] and upregulate complement factor expression [54] of the RPE. The RPE constitutes the outer layer of the blood-retina barrier and plays an essential role in ocular immune regulation and retinal homeostasis. This T cell-mediated modification could lead to different effects, for example, chemotaxis, adhesion, and activation of different cells, such as monocytes and resident ocular microglia.

A hypothesis could be that the combined effect of inflammation and T cell differentiation (accelerated immune aging) initiate drusen. T cells are attracted to the site of tissue damage, inducing further secretion of cytotoxic and pro-inflammatory molecules generating a self-maintaining positive feedback loop. The results in our study indicate that the patients with drusen are the ones with the higher CLI and are the ones with the most accelerated immune aging. These findings could indicate that CLI, accelerated immune aging, or both factors combined could trigger drusen formation or speed the accumulation of debris in the BM.

In a previous study, we found that the RPE-BM complex was significantly thicker in patients with MPNs than an older healthy control group, indicating that the MPN patients accumulated debris and BM thickening earlier, which could be due to their elevated CLI and/or accelerated immune aging. Other factors that induce a pro-inflammatory milieu such as smoking or atherosclerosis and cardiovascular disease also increase the risk of AMD and could therefore be supportive of systemic inflammation being able to initiate drusen formation [1, 55]. The accumulation of chronic inflammatory episodes during a lifetime and how the host’s immune system can control the inflammatory processes may explain why some people develop AMD. The MPN patients show massive inflammation over a longer period and may, as a result, develop drusen.

We also evaluated the MPN biological continuum from early cancer stage ET, over PV, to the advanced PMF stage. In the CD8 compartment, patients with PMF had a significantly higher percentage of terminally differentiated EMRA cells compared to both ET and near significant compared to PV, indicating an increasing senescent profile over the continuum. This fits well with our previous finding of increasing CLI over the continuum [9]. We also observed a tendency to lose costimulatory markers and increased CD56 expression over the continuum, but the results were not statistically significant. This tendency was also observed in the radar plots of the biological continuum, and the PMF patient plots stood more out compared to especially ET patients but also PV patients in some of the plots. Better powered studies could investigate this.

For the MPNs, a concept considers CLI as a trigger and driver of disease development and progression and substantiates the need for early treatment intervention to dampen CLI [56]. This dampening of inflammation may be beneficial as a treatment for AMD patients, potentially decreasing the possible inflammation-driven drusen development and T cell differentiation.

Being an observational study, we can only guess on causality, and further studies using experimental methods are needed.

In conclusion, this and our previous studies suggest that MPNd patients show signs of altered innate immunity (elevated CLI and complement dysregulation) as well as adaptive immunity (accelerated T cell differentiation – more effector T cells/senescent T-cells). These findings may implicate that CLI and senescent T cells play a role in AMD pathogenesis by triggering drusen formation.

## METHODS

### Study design and participants

The participants in this cross-sectional study consisted of the same participants as in our recent work, and the description of the methods in this study will be very similar [9]. We included 29 patients with nAMD, 28 with intermediate AMD (iAMD) [57], and 65 patients with MPNs [58] between July 2018 and November 2020. The MPN participants consisted of two groups, 35 MPNd patients (with drusen corresponding to early or intermediate AMD) and 27 with MPNn. Each participant provided written and oral informed consent after thorough information about the study. The Ethics Committee in Region Zealand, Denmark approved the study, and we adhered to the Helsinki declaration’s tenets. We carried out the study at Zealand University Hospital (ZUH) in Roskilde, and we included and examined patients at the ophthalmology and hematology departments.

The exclusion criteria consisted of patients with other active cancer, inflammatory- or autoimmune diseases, patients receiving immunomodulating treatment (Ruxolitinib, interferon-α), CRP levels >15, and VEGF inhibition therapy within the last eight weeks.

### Retinal imaging and clinical data

The patients had their pupils dilated with tropicamide 1% before obtaining a stereoscopic 45°C color fundus photograph centered on the macula (model TRG-NW8, Topcon). The digital color images were used to identify drusen and determine the patients’ AMD status, using a simplified version of the Wisconsin age-related maculopathy grading system (WARMGS) [14, 59]. Each participant answered a questionnaire about their health status, medication, and lifestyle.

### Blood sampling and flow cytometry

For flow cytometric analyses, we collected venous blood from antecubital veins in ethylenediaminetetraacetic-acid-coated (EDTA) tubes. The same investigator (C.L.) collected blood samples from all patients and performed flow cytometry- cell preparation and analysis within four hours. Leukocyte count was obtained with a Sysmex KX-21NTM (Sysmex Corporation). We obtained 1.0 × 10^6^ leukocytes in the test tube and lysed erythrocytes with a 1% lysis buffer (Nordic BioSite AB). We washed and centrifuged (5 min at 500 × g) the leukocytes three times with BD FACS Flow isotonic buffer (BD Biosciences), and cells were resuspended in isotonic buffer. Hereafter, monoclonal antibodies were added, and cells were incubated for 20 min in the dark at room temperature. Antibodies and fluorochrome-matched negative isotype controls were from R&D Systems; Peridinin-chlorophyll-protein (PerCP) CD4 IgG2a (FAB3791C), Fluorescein Isothiocyanate (FITC) IgG2a (IC0041F); From BioLegend; Phycoerythrin(PE)/Cyanine7(Cy7) CD8a IgG1 κ (300914), Brilliant Violet V510 CCR7 IgG2a (353232), Allophycocyanin (APC) CD56 IgG1 κ (318332), Pacific Blue CD45Ra IgG2b (304123), APC CD28 IgG1 (302912), PE CD27 IgG1 (356406), Brilliant Violet V510 IgG2a (400268), PE/Cy7 IgG1 (400126), APC/Cy7 IgG1 (400128), Pacific Blue IgG2b (400331), APC IgG1 (400120), PerCP IgG2a κ (400250); From Bio-Rad FITC CD45Ro IgG2a (MCA461FT); From BD Biosciences PE IgG1 κ (555749). The fluorochrome-matched negative isotype controls were used for each antibody to identify unspecific binding and were set to a threshold of 1%. In the last step, we washed the stained cells, resuspended them in isotonic buffer, and immediately analyzed them on a flow cytometer (BD FACSCanto II; BD Biosciences). The gating size was 100,000 singlet leukocytes. We used Kaluza Analysis (Kaluza Analysis v. 2.1; Beckman Coulter) to analyze flow data and estimated singlet leukocytes with forward scatter cell height vs. area. We used forward/side scatter area plots to gate lymphocytes and the markers CD4 and CD8 to differentiate CD4+ T cells and CD8+ T cells. Finally, we used the CD45Ra, CD45Ro, CCR7, CD27, CD28, and CD56 expressions and the Boolean function in Kaluza to analyze T cell differentiation, loss of differentiation markers, and CD56 expression.

### Statistical analysis

For data analysis, we used RStudio version 4.1.1. Normally distributed data are shown as mean and 95% confidence interval (CI), non-normal data as median and interquartile range (IQR). We assessed data for normality with histograms and QQ-plots, and we used linear regression or robust linear regression to assess if outcomes were age-dependent. Group comparisons were analyzed for continuous variables with the independent samples *t*-test, Wilcoxon’s rank-sum test, One-way analysis of variance (ANOVA), or Kruskal Wallis test. For categorical values, we used the Chi-squared test or Fisher’s Exact test. Power calculations were based on previous similar immunologic studies of patients with nAMD, with an alpha level of 0.05, a power of 80%, and an effect size of 20%, resulting in a sample size of a minimum of 26 in each group [60, 61]*. P*-values less than 0.05 were considered statistically significant.
